# De Novo Acute Myeloid Leukemia with Combined *CBFB-MYH11* and *BCR-ABL1* Gene Rearrangements: A Case Report and Review of Literature

**DOI:** 10.1155/2020/8822670

**Published:** 2020-12-16

**Authors:** Venkata Rakesh Sethapati, Ra'ed Jabr, Leyla Shune, Wissam El Atrouni, Patrick R. Gonzales, Wei Cui, Shivani Golem

**Affiliations:** University of Kansas Medical Center, Kansas City, KS, USA

## Abstract

Acute myeloid leukemia (AML) with inv(16)(p13.1q22) resulting in *CBFB-MYH11* fusion is associated with a favorable prognosis. The presence of a KIT mutation modifies it to an intermediate prognosis. Additionally, inv(16) can cooperate with other genetic aberrations to further increase cell proliferation. Coexistence of inv(16) and t(9;22) is extremely rare (20 cases). We present a case of a 55-year-old male with elevated white blood cell count. Bone marrow evaluation and flow cytometry analysis were compatible with AML with monocytic features. Cytogenetic studies revealed two-related clones, a minor clone with inv(16) and a major clone with concurrent inv(16) and t(9;22) rearrangements. Fluorescent in situ hybridization studies confirmed these rearrangements. Molecular analysis detected a p190 *BCR-ABL1* transcript protein. *KIT* mutations were negative. The patient was initially treated with standard induction regimen; 7 daily doses of cytarabine from day 1–day 7, 3 daily doses of daunorubicin from day 1–day 3, and 1 dose of Mylotarg (gemtuzumab ozogamicin) on day 1. The detection of t(9;22) led to the addition of daily doses of dasatinib (tyrosine kinase inhibitor) from day 7 onwards. The patient achieved complete remission on day 45. During his treatment course, he acquired disseminated *Fusarium* infection. Day 180 bone marrow evaluation revealed florid relapse with 64% blasts. Cytogenetic study showed clonal evolution of the inv(16) clone with no evidence of the t(9;22) subclone. Eventually, bone marrow transplantation was contraindicated, and the patient was transferred to palliative care. Literature review revealed that AML with co-occurrence of *CBFB-MYH11* and *BCR-ABL1* gene rearrangements was involved by only a small number of cases with de novo and therapy-related AML. Most cases were in myeloid blast crisis of chronic myeloid leukemia (CML). Treatment and prognosis among the de novo AML cases varied and majority of them achieved clinical remission. In contrast, these cytogenetic abnormalities in the blast phase of CML had a poor prognosis. As the prognosis and management of AML is dependent upon the underlying genetic characteristics of the neoplasm, it is imperative to include clinical outcome with such rare combinations of genetic alterations.

## 1. Introduction

Acute myeloid leukemia (AML) is one of the most commonly encountered types of leukemias in adults. AML is characterized by clonal expansion of undifferentiated myeloid blasts and consequentially results in impaired hematopoiesis and bone marrow failure. It is a heterogenous disease clinically, morphologically, and genetically. Cytogenetic aberrations and gene mutations play critical roles in the pathogenesis of AML. There are many driver mutations, and the disease can evolve to accumulate more competing clones.

Core binding factor (CBF) leukemias include t(8;21)/*RUNX1-RUNX1T1* and inv(16)/t(16;16)/*CBFB-MYH11* and are classified as AML with recurrent genetic abnormalities under the World Health Organization (WHO) classification [1, 2]. Inv(16) is found in 5–8% of younger patients with AML and accounts for around 7–10% of all patients with de novo AML [1, 3]. This abnormality results in the fusion of myosin heavy chain 11 gene (*MYH11*) at 16p13 and the core binding factor beta subunit gene (*CBFB*) at 16q12. This gene fusion results in chimeric proteins that suppress transactivation mediated by CBF and lead to impaired hematopoietic differentiation [3]. A bone marrow biopsy typically shows an acute myelomonocytic leukemia, accompanied with atypical eosinophils classified as the FAB subtype M4_Eo_ [3]. Chromosome abnormalities involving CBF rearrangements are associated with a favorable prognosis, except when a *KIT* mutation is present [1].

In addition, genetic alterations can also activate signal transduction pathways and confer a proliferation advantage on hematopoietic cells. *BCR-ABL1* is one such example. *BCR-ABL1* translocation, also known as the Philadelphia (Ph) chromosome, results from a reciprocal translocation between chromosomes 9 and 22: t(9;22)(q34; q11.2). This translocation is necessary in defining chronic myeloid leukemia (CML) and is also responsible for a subset of B cell acute lymphoblastic leukemia (B-ALL) and less commonly in de novo AML [4]. The most common form of *BCR-ABL1* fusion (b2a2 or b3a2) in CML and blast phase of CML results in a 210 kDa product (Major breakpoint), whereas in B-ALL, the main fusion form (e1a2) results in a 190 kDa product (minor breakpoint) [5].

We report a case with combined occurrence of inv(16) and *BCR-ABL1* rearrangements, presenting as de novo AML. Our case also utilized the 190 kDa *BCR-ABL1* fusion protein. With only 20 cases of AML described in literature with similar coexisting inv(16) and *BCR-ABL1* rearrangements, there is a greater need to report such cases.

## 2. Case Presentation

A 55-year-old male presented with worsening fatigue. Upon evaluation, he had leukocytosis (white blood cell count—103 × 10^9^/L), thrombocytopenia (platelets—25 × 10^9^/L), and anemia (hemoglobin—7.6 g/dL). Review of the peripheral blood smear revealed 57% circulating blasts, and flow cytometry was compatible with AML with monocytic features. Myeloblasts comprised 15% of cells and showed expression of CD13, CD33, CD117, CD34, HLA-DR, partial CD123, minimal CD64, CD38, and partial cMPO; the blasts were negative for CD56, CD15, CD11b/C, CD14, TdT, and B and T cell markers. There were around 8% immature monocytic cells present which expressed dim CD45, CD13, CD117, CD33, CD15, partial CD11b, and partial CD64 and were negative for CD34. There were also 14% mature monocytic cells.

**Figure 1:**
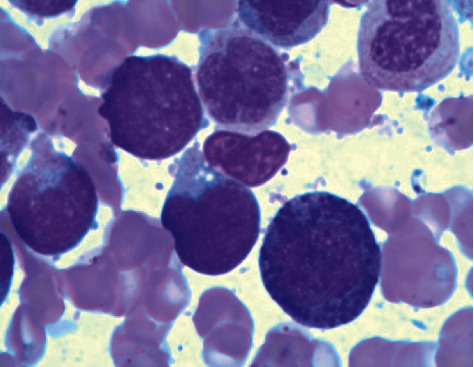
Bone marrow morphology: increased blasts, promonocytes, and atypical eosinophils with large basophilic granules.

A comprehensive bone marrow evaluation was diagnostic for acute myeloid leukemia involving a hypercellular bone marrow (100%) with decreased erythropoiesis, decreased megakaryopoiesis, increased eosinophils, 19% monocytes, and 24% blasts and promonocytes. Morphologically, the blasts appeared large, with irregular and convoluted nuclear contours, dispersed chromatin, and small amounts of basophilic cytoplasm. They were admixed with an increased number of promonocytes that had delicately folded nuclei, dispersed chromatin, inconspicuous nucleoli, and finely granulated cytoplasm. Importantly, there were increased eosinophils that had increased and coarse basophilic granules (Figure 1). Flow cytometry evaluation of the bone marrow biopsy yielded a hemodiluted specimen with myeloblasts expressing CD34, CD13, CD33, CD117, and HLA-DR comprising 1.1% of total cells.

**Figure 2:**
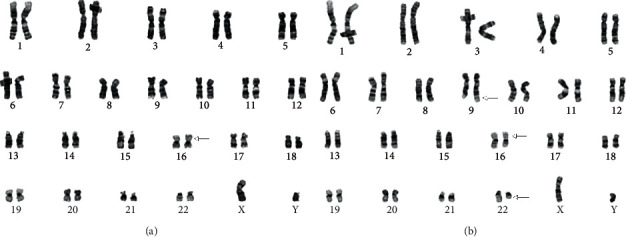
Representative *G*-banded karyogram showing (a) the inv(16)(p13.1q22) as a sole abnormality and (b) co-occurrence of inv(16)(p13.1q22) and t(9;22)(q34; q11.2). The arrows indicate the rearranged chromosomes.

**Figure 3:**
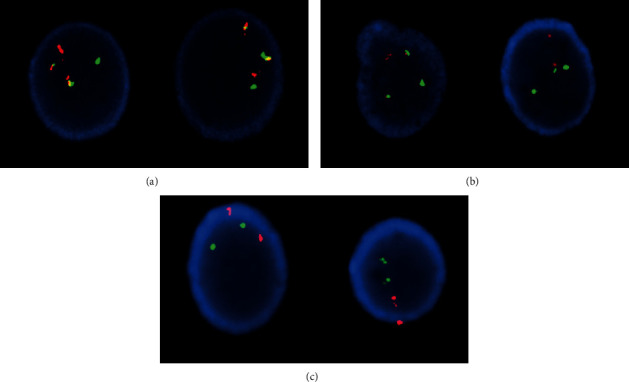
Fluorescence in situ hybridization (FISH) studies on interphase cells showing (a) *CBFB* and *MYH11* rearrangement, dual fusion probe (Cytocell); (b) *BCR/ABL1* translocation, dual fusion probe (Cytocell); and (c) normal FISH signal pattern for *RUNX1T1/RUNX1* rearrangement, dual fusion probe (Abbott molecular).

Conventional cytogenetics at diagnosis demonstrated two-related clones. Two metaphases had pericentric inversions of chromosome 16 (Figure 2(a)). The second related clone was observed in majority of the metaphases, and each had a Philadelphia chromosome with the classic (9;22) translocation along with an inversion 16 abnormality (Figure 2(b)). Fluorescence in situ hybridization (FISH) assays were positive for *CBFB* rearrangement and *BCR/ABL1* translocation in 94.0∼95.0% of the interphase nuclei (Figures 3(a) and 3(b)). A FISH assay for rearrangement of *RUNX1T1/RUNX1* was normal (Figure 3(c)).

A qualitative reverse transcriptase polymerase chain reaction (RT-PCR) was positive for an e1a2 *BCR-ABL1* fusion transcript coding for the 190 kDa *BCR-ABL1* fusion protein. A hematologic neoplasm next generation sequencing (NGS) 141 gene panel that was obtained based on the first bone marrow reported only two mutations of unknown significance in *TET2* (p.L1322R) and *P2RY2* (p.V77A). There were no pathogenic variants detected for *FLT3* exon 14 (internal tandem duplication), *FLT3* exon 20 (tyrosine kinase domain), *NPM1* exon 11, *CEBPA*, and *KIT* exon 17 p.D816V at a minimum allelic fraction of 1.0%.

**Figure 4:**
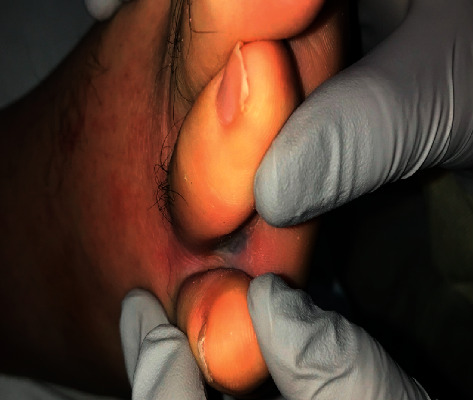
Fusarium infection presenting as a skin ulceration in his toes.

The patient was initially treated with a 7 + 3 cytarabine-based induction regimen: seven daily doses of cytarabine (100 mg/m^2^) from day 1 to day 7, three daily doses of daunorubicin (90 mg/m^2^) from day 1 to day 3, and a single dose of Mylotarg (gemtuzumab ozogamicin, 4.5 mg/m^2^) on day 1. The detection of p190 *BCR-ABL1* transcript at day 5 led to the addition of daily doses of 100 mg of dasatinib (tyrosine kinase inhibitor, TKI) from day 7 onwards. Intrathecal cytarabine therapy was omitted due to a low number of platelets. The patient was pancytopenic and needed multiple red blood cells and platelets transfusions. On day 14, peripheral blood showed severe pancytopenia and bone marrow showed a hypocellular (<5%) bone marrow with decreased trilineage hematopoiesis and 1% blasts. Although flow cytometry evaluation revealed a negative immunophenotypic study, FISH studies were positive for t(9;22) and inv(16) in 25.1–27.1% of the interphase cells. Conventional cytogenetics did not yield enough metaphases for a complete study. Dasatinib was stopped on day 21 due to persistent leukopenia. G-colony stimulating factor was given once on day 21 and on day 23. Notably on day 20, the patient's clinical course was complicated by a skin ulceration in his toes that later resulted in disseminated *Fusarium* infection (Figure 4). Unable to obtain a bone marrow biopsy to evaluate for hematopoietic recovery, dasatinib was restarted on day 34. On day 45, peripheral blood was significant for absolute lymphopenia and monocytosis. Day 45 bone marrow biopsy showed a hypercellular marrow (80%), increased trilineage hematopoiesis, and less than 1% blasts. Flow cytometry on the same specimen was reported as a negative immunophenotypic study. The conventional cytogenetic study showed normal karyotype, and RT-PCR analysis for p190 *BCR/ABL1* transcript was undetectable. His clinical status was in complete remission (CR), and the patient was eventually discharged on day 60.

**Figure 5:**
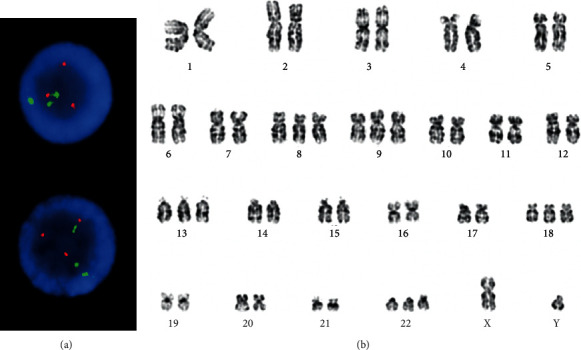
(a) Fluorescence in situ hybridization (FISH) studies on interphase cells showing *BCR* and *ABL1* FISH probe signals, dual fusion probe (Cytocell) and (b) representative *G*-banded karyogram showing relapsed clone of the inv(16)(p13.1q22) primary clone.

Patient was kept on a continued daily dose of 100 mg of dasatinib. However, the patient had gradually increasing Fungitell values and eventually developed fulminant *Fusarium* infection. He was admitted for bilateral pneumonia on day 120 and was treated with amphotericin (5 mg/kg). He was later enrolled on a clinical trial for an alternative oral-based antifungal medication therapy. His fungal infection was reported to have improved with decreased pulmonary infiltrates and a healed foot wound. The patient returned on day 180 for bone marrow transplant evaluation. His peripheral blood smear was remarkable for pancytopenia and 9% circulating blasts. His bone marrow showed persistent AML with 64% blasts and blast equivalents involving a mildly hypercellular bone marrow (50–60%) with decreased trilineage hematopoiesis. The corresponding flow cytometry on the bone marrow specimen revealed 30% of aberrant myeloid blasts that were positive for CD45 (dim), CD34, CD117, HLA-DR, CD13, CD33, and CD38. Chromosome study showed complete resolution of the t(9;22) subclone (negative by FISH and RT-PCR); however, the inv(16) clone resurfaced with other additional abnormalities significant for clonal evolution (Figure 5). Fungitell values were again high, and CT scan showed findings consistent with pneumonia and pansinusitis. Bronchial lavage cultures and blood cultures were negative for microorganism growth. Bone marrow hematopoietic stem cell transplantation (HSCT) was eventually contraindicated. The patient preferred hospice care and died two months after.

## 3. Discussion

An inv(16) abnormality is found in 5–8% of younger patients with AML, while the Ph chromosome is a rare event in AML with a reported incidence ranging from 0.5% to 3% [1, 6]. *BCR-ABL1* AML is now included as a new provisional entity in the 2017 revised WHO classification of hematopoietic malignancies [1, 7]. This entity excludes cases with evidence of the history of CML. Literature review describes a limited number of cases with AML with *BCR-ABL1* translocation that tend to incorporate additional distinct genetic aberrations including inv(16), t(8;21)/*RUNX1-RUNX1T1*, t(15;17)/*PML-RARA*, inv(3), 5q deletion, and *NPM1* mutation [8, 9]. Our case report similarly illustrates a patient with de novo AML with combined *BCR-ABL1* (p190 form) and inv(16) abnormalities.

The Ph chromosome was observed as a subclone in our patient suggestive for “real AML” as Bacher et al. described in his case series [8]. In case #2, their patient had Ph chromosome-positive subclones as a secondary genetic alternation along with inv(16) at initial diagnosis. The patient was treated with hydroxycarbamide and daunorubicin/cytarabine followed by a TKI, imatinib. The patient went into remission but relapsed three months later. At relapse, cytogenetics showed 22 metaphases carrying an inv(16), two of which had an additional t(9;22). The first relapse was treated by intensified chemotherapy using fludarabine, cytarabine, G-CSF, and idarubicin, and again complete remission was achieved. A second relapse occurred ten months from initial diagnosis where 10 of 18 metaphases showed inv(16), while the other eight had evidence of both inv(16) and t(9;22). Their patient's clinical status after the second relapse is unknown. In contrast, our case at relapse showed complete resolution of the t(9;22) subclone (negative FISH and RT-PCR for *BCR-ABL1*), but the inv(16) clone resurfaced with other additional abnormalities significant for clonal evolution. This finding substantiates the efficacy of a TKI inhibitor in cases of Ph-positive AML.

Similar de novo and therapy-related AML cases with concurrent inv(16) and *BCR-ABL1* rearrangements are compared in Table 1 [10–14]. Bustamante and team described a rapid response to single agent treatment with imatinib and demonstrated a negative finding of *BCR-ABL1* fusion by FISH or RT-PCR after 3 weeks of therapy. Salem et al. included one patient with de novo AML and one with initial AML with *CBFB* rearrangement but subsequently acquired a *BCR-ABL1* translocation [4]. The former received induction therapy with FLAG-IDA (fludarabine, cytarabine, idarubicin, and G-CSF) regimen and dasatinib with clinical remission in 21 months, and the latter received an induction with 7 + 3 regimen and dasatinib but relapsed after 8 months and died 2 years after initial diagnosis [4]. Miura et al. and Secker-Walker et al. reported stable remissions up to 70 months were achieved after allogenic stem cell transplantation.

We also know that the Ph chromosome can pair up with other abnormalities in AML. Han JY and team presented an AML case with a known prognostically adverse aberration inv(3) and monosomy 7 with Ph chromosome as a secondary abnormality at diagnosis [15]. The patient was treated with induction chemotherapy, but the patient's course was complicated by neutropenic fever and acute renal failure. Repeated bone marrow examinations showed the presence of persistent leukemia, and the patient subsequently went into hospice care. Mozziconacci et al. described two cases, one in which *BCR-ABL1* translocation was in combination with inv(3) where the patient did not respond to initial treatment and died [9]. The other case was a more favorable rearrangement involving acute promyelocytic leukemia where a total of 100 mitoses were analyzed and two metaphases with t(15;17) and t(9;22) were found. The patient was treated with all‐trans retinoic acid and chemotherapy and achieved complete remission on day 30. At 3 months, a very low level of MBCR (b2a2) transcript was detected. RT-PCR was still positive at 4 months but negative at 9, 14, 17, and 30 months. 4 years after diagnosis, the patient was still in remission. These findings suggest prognosis of AML with Ph-positive subclones can be associated with the corresponding cytogenetic abnormality.

Interestingly, cases with CML in the blast phase that acquire an inv(16) aberration have a poor prognosis. Salem et al. studied ten patients with combined inv(16) and *BCR-ABL1* cytogenetic abnormalities and seven of which were CML cases [4]. In the seven patients, six died with a median overall survival time of 14 months despite intensive chemotherapy and targeted therapy with TKI [4]. Analyzing these cases and cases from Table 1, we can conclude that the p210 form was found in CML cases that transformed into the blast phase, and the p190 form was found in ‘true AML' with the exception of the case by Ninomiya et al. [16]. The p190 isoform is also more commonly associated with leukemogenesis leading to *BCR-ABL1*-positive acute lymphoblastic leukemia than AML [17, 18]. However, the immunophenotypic study from our case is against a lymphoblastic or mixed phenotypic leukemia.

Due to the rarity of this disease, the prognosis is difficult to determine. *CBFB* rearrangement in AML with no additional mutation is known to have a favorable prognosis. A study with 201 de novo AML patients indicated that the 5-year survival probability was 43% for *CBFB-MYH11* rearrangements [19]. Ph-positive AML appears to be an aggressive disease with poor response to traditional AML therapy or TKI alone [1, 4]. Additionally, only limited cases in literature included multigene mutation analysis in these rare co-occurring inv(16) and *BCR-ABL1* abnormalities. Our case and the study by Salem et al. did not detect any pathogenic variants. This indicates that these co-occurring abnormalities act independently of other commonly seen mutations in myeloid neoplasms. Our patient had initially shown a favorable response to treatment and achieved CR by day 45 postdiagnosis. He later developed disseminated *Fusarium* infection, which compromised his leukemic treatment. Incidence of *Fusarium* spp. infection in patients with acute leukemia is 0.06% and the survival rate is 4% [20]. Despite enrolling in an investigational antifungal clinical trial, his fungal infection persisted and was not a suitable candidate for HSCT. Overall, we feel that the combination of inv(16) and *BCR-ABL1* genetic abnormalities confers an intermediate prognosis.

De novo AML patients with combined inv(16) and t(9;22) cytogenetic abnormalities seem to benefit from intensive chemotherapy and targeted TKIs. Decisions about HSCT in intermediate-risk AML were less clear-cut in the past, and nowadays, most patients are considered for HSCT in their first CR [21]. Patient fitness, availability of a sibling donor or an alternative donor, a clinical trial option, and the transplant center experience must be considered when making a decision about HSCT. It is important to note that a longer duration of *BCR-ABL1* fusion transcript surveillance can be used to establish a standard of care for such Ph-positive AML patients with additional rearrangements [7].

## 4. Conclusion

Molecularly defined genetic abnormalities in AML have a significant impact on a patient's management and prognosis. AML presenting with both inv(16) and t(9;22) abnormalities confers an intermediate prognosis. The pathognomic effect of the occurrence of these abnormalities acts independent of other common molecular mutations seen in myeloid neoplasms. These cases are rare and need to be documented and studied further to formulate better management strategies. Such cases may benefit from intensive chemotherapy and TKIs as a bridge to stem cell transplantation. Comorbidities and infectious diseases arising from an immunocompromised state must be taken into consideration when planning treatment regimens to prevent doing more harm than benefit to the patient.

## Figures and Tables

**Table 1:** AML cases with concurrent inv(16) and BCR-ABL genetic abnormalities in this study and selected cases from literature review.

Sex/age	AML subtype	FAB classification	Karyotype	Other mutation assay(s)	Clinical course	PMID
M/40	De novo	M4eo	46,XY,inv(16)(p13q22)[17]/46,XY,idem,t(9;22)(q34:q11)[3]	Not performed	Chemotherapy and HSCT; stable remission	8250017
F/9	De novo	M4eo	46,XX,inv(16)(p13q22)[21]/46,XX,t(9;22)(q34;q11),inv(16)(p13q22)[8]/46,XX [10]	Not performed	Chemotherapy and HSCT; died soon after HSCT	1728947
M/13	De novo	M4	46,XY,inv(16)(p13.1q22)[2]/46,idem,del(7)(q22q32)[16]/46,idem,t(9;22;19)(q34;q11.2;p13.1)[2]	Not performed	Cytarabine, daunomycin, etoposide + gemtuzumab	20513535
F/40	De novo	M1	46,XX,inv(16)(p13q22)[4]/46,XX,t(9;22)(q34;q11),inv(16)(p13q22)[18]	Not performed	One cycle of conventional induction therapy	11368385
M/63	De novo	M4eo	At diagnosis: 46,XY,inv(16)(p13q22)[20]/46,XY,t(9;22)(q34;q11),inv(16)(p13q22)[2] At last relapse: 46,XY,inv(16)(p13q22)[10]/46,XY,+8,t(9;22)(q34;q11),inv(16)(p13q22)[8]	Not performed	Hydroxycarbamide, daunorubicin/ cytarabine and imatinib with relapse in 3 months. Fludarabine, cytarabine, G-CSF and Idarubicine with complete remission but relapsed 10 months later	21275954
F/49	Therapy- related	M4eo	46,XX,inv(16)(p13.1q22)[5]/46,XX,t(9;22)(q34;q11.2)[7]/46,XX[8]	Not performed	Imatinib; remission in 3 weeks	22370710
M	De novo	M4eo	46,XY,inv(16)(p13.1q22)[3]/46,idem,t(9;22)(q34;q11.2)[17]	None (NGS, multiple genes)	Fludarabine, cytarabine, G-CSF and Idarubicine + dasatinib; stable remission	28253536
M/55	De novo	M4eo	At Diagnosis: 46,XY,inv(16)(p13.1q22)[2]/46,sl,t(9;22)(q34;q11.2)[20]/46,XX[1].nuc ish(MYH11,CBFB)x3(MYH11 con CBFBx2)[190/200]/(ABL1,BCR)x3(ABL1 con BCRx2)[188/200] 13-day follow-up: 46,XY[2].nuc ish(ABL1,BCR)x3(ABL1 con BCRx2)[88/350]/(MYH11,CBFB)x3(MYH11 con CBFBx2)[95/350] 45-day follow-up: 46,XY[20] 188-day follow-up: 51,XY,+8,+9,+13,inv(16)(p13.1q22),+18,+22[19]/46,XY[1].nuc ish(MYH11,CBFB)x3(MYH11 con CBFBx2)[155/200]/(ABL1x2∼3,BCRx2∼3)[372/500]	None (NGS, multiple genes)	Gemtuzumab ozogamicin induction + Dasatinib; a first relapse diagnosed 6 months from initial diagnosis. Hospice.	Current case

## Data Availability

The data used to support the findings of this study are included within the article.
